# Renal Sinus Fat Is Expanded in Patients with Obesity and/or Hypertension and Reduced by Bariatric Surgery Associated with Hypertension Remission

**DOI:** 10.3390/metabo12070617

**Published:** 2022-07-02

**Authors:** Emilia Moritz, Prince Dadson, Ekaterina Saukko, Miikka-Juhani Honka, Kalle Koskensalo, Kerttu Seppälä, Laura Pekkarinen, Diego Moriconi, Mika Helmiö, Paulina Salminen, Pirjo Nuutila, Eleni Rebelos

**Affiliations:** 1 Turku PET Centre, University of Turku, 20520 Turku, Finland; emilia.k.moritz@utu.fi (E.M.); pryada@utu.fi (P.D.); mjhonk@utu.fi (M.-J.H.); kerttu.seppala@utu.fi (K.S.); laura.pekkarinen@tyks.fi (L.P.); pirjo.nuutila@utu.fi (P.N.); 2 Department of Radiology, Turku University Hospital, 20521 Turku, Finland; ekaterina.saukko@tyks.fi; 3 Department of Medical Physics, Turku University Hospital, 20521 Turku, Finland; kalkos@utu.fi; 4 Department of Endocrinology, Turku University Hospital, 20521 Turku, Finland; 5 Department of Surgical, Medical, Molecular Pathology and Critical Care Medicine, University of Pisa, 56124 Pisa, Italy; d.moriconi@ao-pisa.toscana.it; 6 Division of Digestive Surgery and Urology, Turku University Hospital, 20521 Turku, Finland; mika.helmio@tyks.fi (M.H.); paulina.salminen@tyks.fi (P.S.); 7 Department of Surgery, University of Turku, 20520 Turku, Finland; 8 National Research Council, 56124 Pisa, Italy

**Keywords:** renal sinus fat, obesity, bariatric surgery

## Abstract

Renal sinus fat is a fat depot at the renal hilum. Because of its location around the renal artery, vein, and lymphatic vessels, an expanded renal sinus fat mass may have hemodynamic and renal implications. We studied whether renal sinus fat area (RSF) associates with hypertension and whether following bariatric surgery a decrease in RSF associates with improvement of hypertension. A total of 74 severely obese and 46 lean controls were studied with whole-body magnetic resonance imaging (MRI). A total of 42 obese subjects were re-studied six months after bariatric surgery. RSF was assessed by two independent researchers using sliceOmatic. Glomerular filtration rate (eGFR) was estimated according to the CKD-EPI (Chronic Kidney Disease Epidemiology Collaboration). Patients with obesity accumulated more RSF compared to lean controls (2.3 [1.7–3.1] vs. 1.8 [1.4–2.5] cm^2^, *p* = 0.03). Patients with hypertension (N = 36) had a larger RSF depot compared to normotensive subjects (2.6 [2.0–3.3] vs. 2.0 [1.4–2.5] cm^2^, *p* = 0.0007) also after accounting for body mass index (BMI). In the pooled data, RSF was negatively associated with eGFR (*r* = −0.20, *p* = 0.03), whereas there was no association with systolic or diastolic blood pressure. Following bariatric surgery, RSF was reduced (1.6 [1.3–2.3] vs. 2.3 [1.7–3.1] cm^2^, *p* = 0.03) along with other markers of adiposity. A total of 9/27 of patients achieved remission from hypertension. The remission was associated with a larger decrease in RSF, compared to patients who remained hypertensive (−0.68 [−0.74 to −0.44] vs. −0.28 [−0.59 to 0] cm^2^, *p* = 0.009). The accumulation of RSF seems to be involved in the pathogenesis of hypertension in obesity. Following bariatric surgery, loss of RSF was associated with remission from hypertension.

## 1. Introduction

In the last decades, we have been facing a global epidemic of obesity, with the prevalence of the disease having tripled in the last four decades [1]. Currently, more than 1.9 billion people are overweight and over 650 million people are obese [1]. Obesity leads to a myriad of metabolic complications: type 2 diabetes (T2D), hypertension, dyslipidemia, cardiovascular disease, chronic kidney disease (CKD), neurodegenerative diseases, asthma, musculoskeletal disorders, and higher vulnerability to infections, as well as an increased risk for many types of cancers [2,3,4,5,6,7,8]. 

Arterial hypertension is directly related to obesity in at least 75% cases [6]; vice versa, up to 50% of obese individuals may suffer from arterial hypertension [7]. Hypertension is a well-established risk factor for end-stage renal failure [9]. Together with renin–angiotensin–aldosterone system (RAAS) activation, renal venous hypertension increases glomerular and renal interstitial hydrostatic pressure leading to decreased net filtration pressure and glomerular filtration rate (GFR) [7]. 

Renal sinus is the perirenal hilum region at the medial border of the kidney where the renal artery, the renal vein, lymphatic vessels, and the ureter enter the kidney. The expansion of the renal sinus fat mass, which is a part of visceral adipose tissue (VAT), has been associated with both higher systolic blood pressure or the higher number of antihypertensive drugs needed [10,11], as well as to decreased GFR [10,12] and microalbuminuria [13]. Mechanistically, excessive fat accumulation in the renal sinus would result in increased intra-abdominal pressure and compression of the low-pressure renal venous structures [14,15], with resulting alteration of the renal hemodynamics, possibly by activation of the RAAS [15]. Thus, the expansion of the renal sinus fat mass seems to be involved in the further deterioration of hypertension and renal dysfunction in patients with obesity. Although most dietary interventions fail, bariatric surgery is currently the most effective means to inducing good and sustained weight loss [16] and long-lasting remission of T2D [17] even though it is not exempt from complications, such as postprandial hypoglycaemia [18,19] and nutritional deficiencies [20,21]. Bariatric surgery has also been shown to improve renal function and contribute to a significant reduction in blood pressure [22]. However, thus far it has not been studied whether weight loss following bariatric surgery leads to a reduction in the renal sinus fat mass and whether such a decrease may be related to improvements in renal and hypertension outcomes following significant surgery-induced weight loss. Therefore, in this study, we assessed whether renal sinus fat area (RSF) associates with hypertension and whether following bariatric surgery a decrease in RSF associates with the improvement of hypertension. 

## 2. Results

Patients with obesity and healthy lean controls were well-matched in terms of age, but as expected patients affected by obesity had higher adiposity measures comprising of subcutaneous adipose tissue (SAT) and visceral adipose tissue (VAT) fat mass, higher systolic and diastolic blood pressure, worse insulin sensitivity, and higher inflammatory markers. Estimated glomerular filtration rate (eGFR) expressed in ml/min/1.73 m^2^ was not different between the two groups, but when accounting for the individual BSA, total eGFR (mL/min) was higher in patients with obesity. The anthropometric and biochemical characteristics of the study participants are listed in Table 1.

### 2.1. RSF

**Figure 1:**
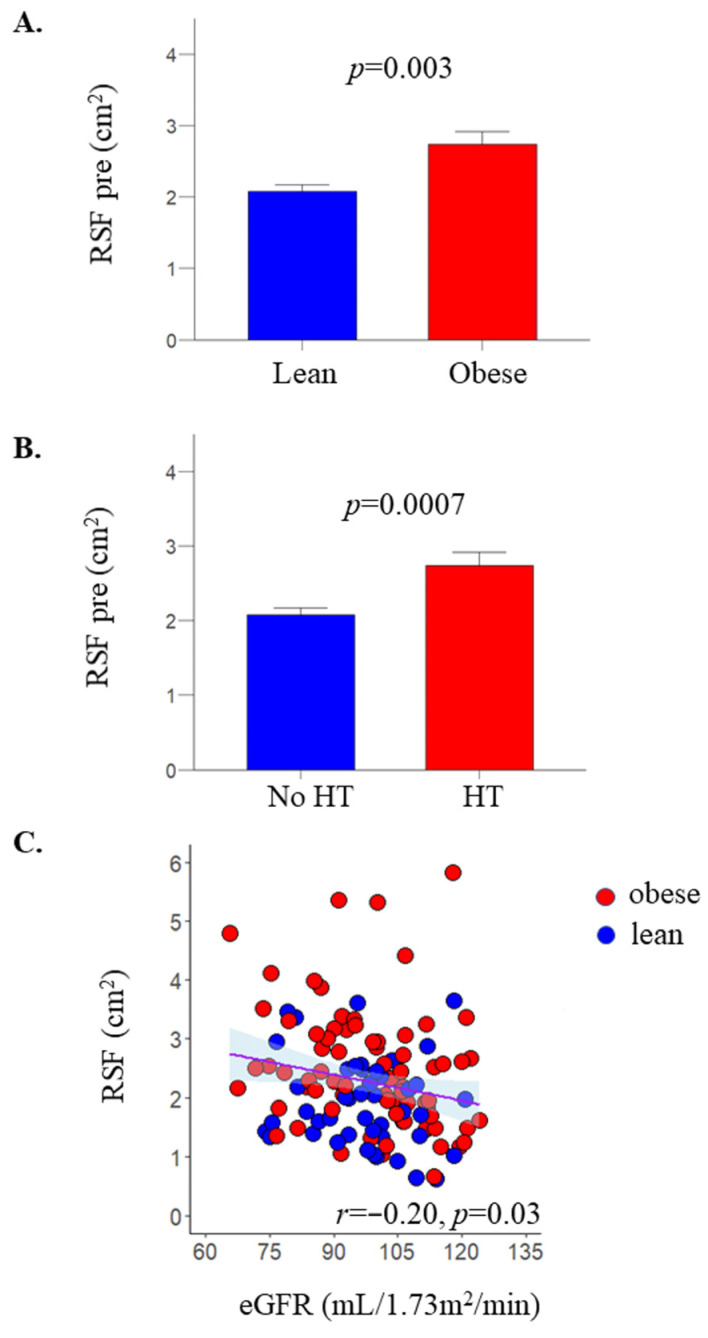
RSF was higher in patients with obesity, compared to healthy lean subjects (**A**), and also in patients with hypertension (HT) compared to subjects without hypertension (**B**). Data were mean ± SE. In the pooled data, RSF correlated inversely with estimated glomerular filtration rate (eGFR) (**C**).

In line with the expanded VAT mass, patients with obesity also had larger accumulations of fat in the (RSF) compared to the lean controls (Table 1). In the pooled data, RSF was associated with VAT (*r* = 0.53, *p* < 0.0001) and to a smaller extent with SAT mass (*r* = 0.20, *p* = 0.04), age (*r* = 0.29, *p* = 0.001), and BMI (*r* = 0.26, *p* = 0.001). In line with these findings, patients with obesity had higher RSF compared to lean controls (2.3 [1.7 to 3.1] vs 1.8 [1.4 to 2.5] cm^2^, *p* = 0.003) (Table 1 and Figure 1A). Men also had larger RSF compared to women (3.1 [2.6 to 3.6] vs. 2.0 [1.5 to 2.6] cm^2^, *p* = 0.0001). RSF was not associated with systolic or diastolic blood pressure. However, on dividing the study population in patients with and without hypertension, RSF was significantly higher in patients with hypertension (Figure 1B), and this effect remained significant also when accounting for BMI (*p* = 0.02). RSF was negatively associated with eGFR (*r* = −0.20, *p* = 0.03) (Figure 1C). 

### 2.2. After Bariatric Surgery 

After bariatric surgery patients achieved significant weight loss (they lost on the average ~23% of their initial body weight) and blood pressure was improved (Table 1). A total of 1 out of 3 patients with hypertension (9/27) at baseline achieved remission of hypertension after surgery, while 15/42 patients remained normotensive. Moreover, their glucometabolic status and insulin sensitivity were improved. Of the 20 patients with T2D who were studied both before and after bariatric surgery, 45% of them (9/20) achieved remission from the disease.

**Figure 2:**
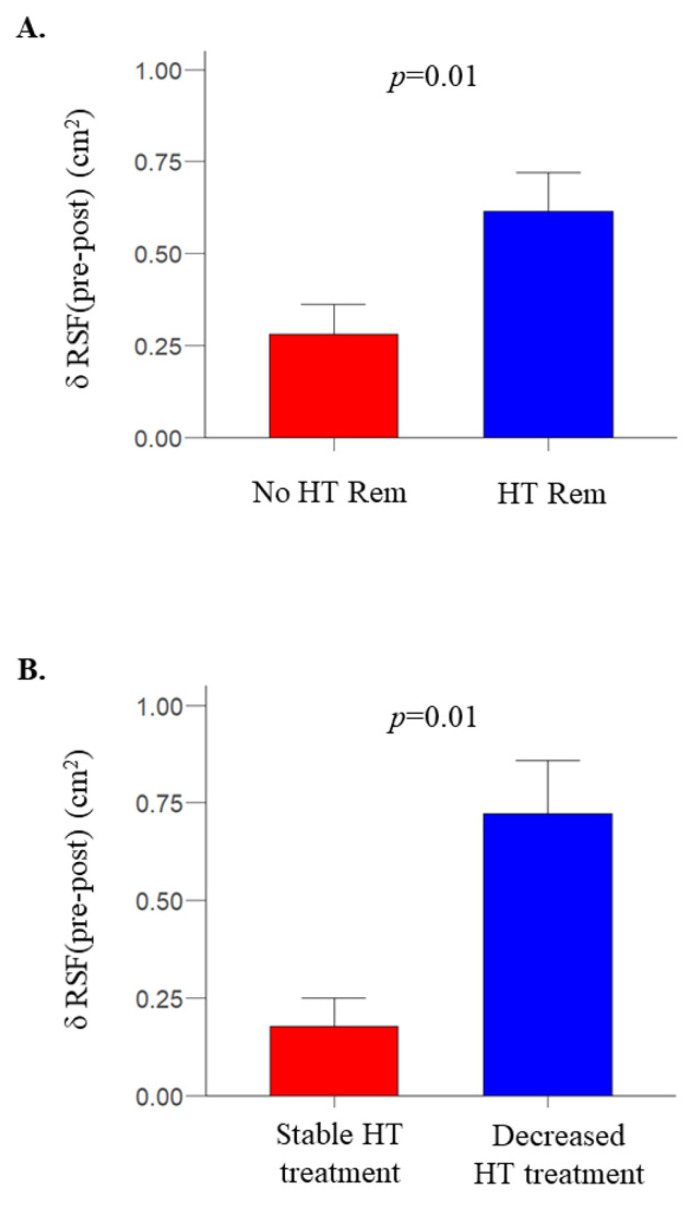
Change in RSF was larger in patients who achieved remission from hypertension following bariatric surgery compared to non-remitters (**A**). In the non-remitters group, change in RSF was larger in patients who decreased the number of antihypertensive drugs used compared to those who remained on stable antihypertensive medication (**B**). Note that in the “decreased HT treatment” group (panel **B**), data of only 3 subjects were available, which may explain the apparently larger change in RSF in these few subjects compared to the HT Rem group of panel (**A**). Data were mean ± SE.

RSF was significantly decreased following bariatric surgery (2.3 [1.7–3.1] vs. 1.6 [1.3–2.3] cm^2^, for before and after bariatric surgery, respectively, *p* < 0.0001); as were other fat depots (Table 1). The change in RSF was not associated with the change in systolic, diastolic, or mean arterial blood pressure. However, in an ad hoc analysis, in patients who had hypertension at baseline and were divided by hypertension remission outcome following bariatric surgery, change in RSF was greater in patients who achieved remission compared to those that did not achieve remission (−0.68 [−0.74 to −0.44] vs. −0.28 [−0.59 to 0] cm^2^, respectively, *p* = 0.01) (Figure 2A). Moreover, in patients with no hypertension remission (N = 17), the change in RSF was larger in patients who decreased the number of antihypertensive drugs (N = 3) compared to those who were on the same number of drugs as it was before the intervention (N = 14) (−0.65 [−0.99 to −0.54] vs. −0.21 [−0.33 to −0.11] cm^2^, *p* = 0.01) (Figure 2B). 

## 3. Materials and Methods

### 3.1. Participants and Study Design 

**Figure 3:**
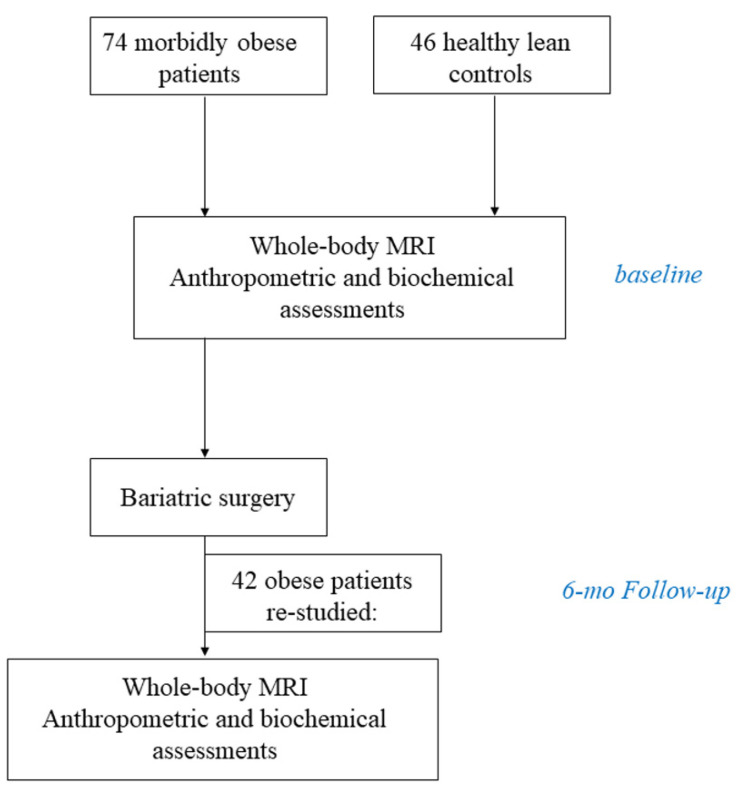
Flow chart of the study.

Data of three clinical studies were analysed, where the main topic was to assess differences in tissue metabolism between obese and lean individuals and the effect of bariatric surgery-induced weight loss on whole-body insulin sensitivity and tissue metabolism [23,24,25]. Whereas subjects were studied with both positron emission tomography and whole-body MRI, in the present analysis, only the MRI data were used and analysed. The dataset comprised of 74 patients with morbid obesity and 46 lean controls. Inclusion and exclusion criteria and the surgical techniques have been previously described in detail [26]. In brief, patients with obesity who were referred to the Turku University Hospital for bariatric surgery were recruited. The inclusion criteria were BMI > 40 kg/m^2^ or >35 kg/m^2^ with an additional risk factor, aged 18–60 years, and a history of non-successful carefully planned conservative treatments. Individuals using insulin treatment and/or with mental disorders, eating disorders, excessive use of alcohol, or poor compliance were excluded, as were those with a body weight over 150 kg because of restrictions of the imaging devices. Healthy lean participants were recruited via an advertisement in local newspapers. Thirty-six patients with obesity had arterial hypertension, which is defined as arterial blood pressure greater than 140/90 mmHg, use of antihypertensive medication to lower blood pressure [27], or previous diagnosis of hypertension. All lean subjects had normal blood pressure. Forty-two patients with obesity underwent bariatric surgery (19/42 underwent gastric bypass, and the rest laparoscopic Sleeve gastrectomy) and were re-studied with basic anthropometric and biochemical studies and also with a standard 75 g oral glucose tolerance test and whole-body MRI six months after the intervention. Lean subjects were studied with the same evaluations once (Figure 3). Remission from hypertension was defined as normal blood pressure levels without the need for antihypertensive medication at the follow-up visit [28]. Change in number of antihypertensive drugs used was also assessed. The study protocols were approved by the Ethics Committee of the Hospital District of Southwest Finland, and all subjects gave their written informed consent before participating in the study (NCT00793143; studies performed from March 2009 to October 2010, NCT01373892; studies performed from March 2011 to October 2013 and NCT04343469; studies performed from February 2019 to June 2021).

### 3.2. Study Protocol

Clinical screening and anthropometric and biochemical measurements were performed as previously described [26]. Blood pressure was measured with OMRON 711 automatic blood pressure monitor (Omron Corporate, Kyoto, Japan). Before the measurements, subjects were sitting for >10 min in a quiet room. A study nurse then assessed each subject twice for blood pressure measurements within a five-minute interval, and the average value was considered for the analysis. Subjects then underwent whole-body magnetic resonance imaging (MRI) with either a Philips Gyroscan Intera 1.5 T CV Nova Dual scanner (Philips, Amsterdam, The Netherlands) or with a Siemens Magnetom Skyra fit 3T MRI scanner (Siemens Medical Solutions, Erlangen, Germany). MRI acquisition was performed with axial T1-weighted dual fast field echo images (echo time (TE) 2.3 and 4.6 ms, repetition time (TR) 120 ms, slice thickness 10 mm without gap, matrix 256 × 256) or with T1-weighted 3D VIBE two-point DIXON sequence in breath-hold mode (TE 1.2 and 2.5 ms, TR 4.0 ms, slice thickness 2 mm with 0.4 mm gap, matrix 182 × 224). Subjects were scanned from head to knee or to ankle in a supine position. Total scan duration was 20 min. In obese patients, the imaging studies were performed before the standard four-week very-low calorie diet that preceded surgery. 

### 3.3. Distribution of Body Fat

**Figure 4:**
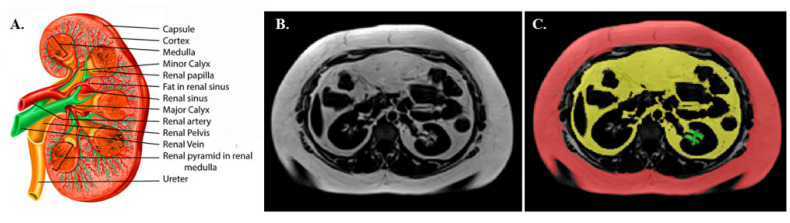
Sketch of the renal anatomy (Source: [29] distributed under the terms of the Creative Commons Attribution 4.0 International License (http://creativecommons.org/licenses/by/4.0/, accessed on the 6 May 2022) © 2022, StatPearls Publishing LLC, (**A**). Representative examples of an MRI (**B**) and RSF measurement (green), SAT (red) and VAT (yellow) volume (**C**). For RSF, a single MRI slice approach was followed. RSF was manually traced, excluding the renal artery and vein from the RSF measurements.

Abdominal fat volumes were calculated by one user (P.D.) from a whole-body MRI scan (Gyroscan Intera CV Nova Dual; Philips, Amsterdam, the Netherlands, or 3T Skyra, Siemens) using the SliceOmatic Tomovision software (version 4.3) as previously reported [23] (Figure 4C).

### 3.4. Renal Sinus Fat Area (RSF) Determination 

Figure 4A provides a sketch of the renal anatomy. A single MRI slice of RSF measurement was done as previously described [10,12]. After visual inspection of the whole kidney area, the areas of renal sinus fat on both kidneys were identified by using anatomic landmarks and were manually segmented within the curvature of the kidney, excluding the renal vasculature and the renal collecting system (Figure 4B,C). Left and right RSF values are shown in Table 1, and then the two measurements were averaged and were used in the analyses (average RSF = (left RSF + right RSF)/2).

### 3.5. Estimated Glomerular Filtration Rate (eGFR) 

The eGFR was calculated by the Chronic Kidney Disease Epidemiology Collaboration (CKD-EPI) equation [30].

### 3.6. Body Surface Area (BSA) 

BSA was estimated as previously described by Du Bois and Du Bois; a formula using each individual’s body weight and height [31].

### 3.7. Oral Glucose Insulin Sensitivity (OGIS) 

OGIS was calculated from the oral glucose tolerance test data, as previously described by Mari et al. [32].

### 3.8. Analytical Methods 

Glycosylated hemoglobin (HbA1c) was measured with ion-exchange high performance liquid chromatography (Variant II Haemoglobin A1c, Bio-Rad Laboratories, California, USA) or a photometric immunoturbidimetric method (Tina-quant Hemoglobin A1c Gen 3, Cobas c501, Roche Diagnostics GmbH, Mannheim, Germany). Plasma insulin and C-peptide were determined by automatized electro-chemiluminescence analyser immunoassay (Modular E170, Roche Diagnostics GmbH, Mannheim, Germany) [33]. Plasma glucose, total cholesterol, HDL-cholesterol, triacylglycerols, and creatinine were measured with a photometric, enzymatic method (Modular P800 or Cobas c702, Roche Diagnostics GmbH, Mannheim, Germany) and LDL-cholesterol using the Friedewald formula [34] or automatised enzymatic assay (Cobas c702, Roche Diagnostics GmbH, Mannheim, Germany). Serum high-sensitivity C-reactive protein (hs-CRP) was analysed using immunonephelometry. Detailed information regarding the analytical methods used can be found on the official laboratory services handbook of Turku University Hospital laboratory units (http://webohjekirja.mylabservices.fi/TYKS/, accessed on 5 May 2022). 

### 3.9. Statistical Analysis

Data were presented as mean ± SD (or median [IQR] for non-normally distributed variables). Categorical variables were expressed as percentages. The comparison between categorical variables was performed by the chi^2^ test. Group comparisons were performed with Student’s t-test for normally distributed or the Wilcoxon rank test for non-normally distributed variables. Associations were tested with a linear logistic regression analysis. Statistical analyses were done using JMP version 13.0 (SAS Institute, Cary, NC, USA). A *p* value < 0.05 was considered statistically significant. Plots were created using the packages ggplot2 and psych of the R statistical computing environment version 4.1.1 (10 August 2021), created from the R Core Team (Vienna, Austria) and Rstudio (version 1.4.1717) [35,36]. To assess reliability of measurement, 30 cases were independently analysed by another operator (E.S.), blind to the assessment by the other reader (E.M.). We assessed interrater reliability by variability (absolute difference between measurements by two readers, divided by the average of those measurements), and intraclass correlation coefficients (ICC). Whilst there are no generally accepted criteria about interpretation of ICC values, we used the suggestion from Portney and Watkins, where <0.5 is poor, 0.5–0.75 is moderate, 0.75–0.9 is good, and >0.9 represents excellent reliability as a guideline [37]. We found interrater variability (13% for the right renal fat measurement and 12% for the left) and an ICC estimate of 0.91 for the right-side measurements and 0.86 for the left-side measurements, suggesting good to excellent agreement. 

## 4. Discussion

This study yielded several outcomes. First, patients with obesity accumulate more fat in the renal sinus compared to heathy lean individuals. Patients with hypertension also have larger RSF, compared to normotensive subjects. In the pooled data, renal sinus fat correlates inversely with eGFR. Following bariatric surgery, RSF was decreased and patients who achieved hypertension remission had a larger decrease in RSF compared to patients who did not achieve remission. Finally, in patients with no hypertension remission post-bariatric surgery, a larger decrease in RSF was associated with a decrease in the number of antihypertensive drugs used. 

RSF is a compartment of VAT that is situated in the renal hilum. Along with the well-established expansion of VAT in patients with obesity, RSF is also expanded, with the two measurements being directly and strongly related. VAT accumulation has been shown to be related to worse cardiologic burden [38]. Metabolic associated fatty liver disease (i.e., ectopic liver fat accumulation) is also associated with a ~1.6-fold increased risk of developing hypertension [39]. We found that patients with hypertension had an expanded RSF—independently of BMI. This finding is in line with previous studies, which have also shown that an expanded RSF is associated with hypertension, or with the number of antihypertensive drugs needed [10,11]. However, we did not detect any association between systolic, diastolic, or mean arterial pressure values and RSF, as previously reported [12]. This may be attributed to the fact that our study population was comprised of both healthy lean controls and morbidly obese patients with hypertension who were already on antihypertensive treatment. The expected blood pressure lowering effect of the antihypertensive drugs could thus have masked the presence of positive correlation between RSF and blood pressure values. 

In a subset of the Framingham cohort, Foster and colleagues demonstrated that “fatty kidney” (defined as RSF greater than the 90th percentile of a nonobese group) was associated with a higher odds ratio of hypertension and CKD—independently of BMI or VAT [10]—demonstrating the specificity of RSF in the development of hypertension and CKD [10]. Even though the exact mechanisms linking RSF expansion and hypertension or CKD were not studied, the authors suggested a plausible mechanism according to which the compression of the renal veins by the accumulated fat could lead to sodium retention and hypertension. 

Following bariatric surgery, the median decrease in BMI was 10 kg/m^2^. As expected, SAT and VAT depots were also largely decreased. We found that RSF was also significantly decreased. This finding contrasts the results of Krievina et al. who have previously reported that significant reduction in VAT (>5% decrease) is not associated with a significant reduction in renal sinus fat mass [40]. That study was an observational prospective study on patients with a BMI ranging from 18–35 kg/m^2^, and the only intervention was participants receiving daily text messages with practical recommendations on how to balance caloric intake and physical activity to achieve and maintain a healthy body weight. The smaller baseline BMI of the subjects and the smaller weight loss achieved may have hampered the study from detecting a change in RSF mass. 

The most salient finding of the present study was that following bariatric surgery, not only was the amount of RSF decreased, but more importantly, subjects who achieved remission from hypertension after bariatric surgery also had a larger decrease in RSF. In keeping with this result, patients with no hypertension remission who achieved reduction in antihypertensive drugs use had a larger decrease in RSF compared to those who maintained the same drugs following bariatric surgery. Ricci et al. assessed perirenal fat thickness by ultrasound. They reported that following sleeve gastrectomy, patients decreasing the number of antihypertensive drugs had higher perirenal fat thickness at baseline [41]. However, in that study perirenal fat mass was assessed only before the intervention, and thus it remains unclear whether the change in perirenal fat mass was associated with the change in the number of drugs needed or with remission from hypertension. Taken together, the close relationship between RSF and hypertension both cross-sectionally and following a weight loss intervention further underlines the finding that the accumulation of fat in this specific visceral depot may be linked with the pathophysiology of hypertension in obesity. Obesity is characterized by expanded blood volume and increased cardiac output [42]. An activated RAAS seems like a plausible mechanism for the expanded blood volume in patients with obesity [43], possibly triggered by compression of the renal vein from the adjacent fat depot. Following weight loss, both plasma renin activity and plasma aldosterone levels have been shown to decrease [44]; thus, it would be highly informative if future studies could assess whether RSF associates with plasma renin activity and plasma aldosterone levels before and after weight loss. 

The strength of the present study is the measurement of RSF both at baseline and after bariatric surgery, using state-of-the art methods, such as MRI. Our study also has some limitations. First, the number of patients studied following bariatric surgery was rather small; future studies are needed to confirm the present findings. Since our dataset consisted predominantly of women the generalizations of the findings to a population consisting of men need to be done with caution. This is of particular importance since men tend to accumulate more fat in the visceral depot, as discussed in [45], and as shown in the present study. In addition, it would have been of interest to assess whether renal sinus fat and its change following weight loss associate with albuminuria and with the measured rather than the estimated glomerular filtration rate, but these measurements were not performed in the present study. Future studies are thus warranted to assess the impact of RSF decrease following weight loss on renal outcomes. Finally, the present RSF values were somewhat larger compared to those reported in the large study by Foster and colleagues [10]. In that study, a BMI > 30 kg/m^2^ was an exclusion criterion, so the more expanded RSF in the present study may be attributed to the inclusion of patients with severe obesity. However, RSF in the lean controls was also larger in our study compared to the no fatty kidney group of the study by Foster et al., even though the authors mentioned that RSF ranged from the lower limit of detection (0.0048 cm^2^) to 4.89 cm^2^—an upper limit similar to our range (0.62–5.83 cm^2^). We think that the discrepancy, mainly at the low end values, may be related to technical issues (for instance to the selection of a different slice for the drawing of the RSF area, or to differences in the thickness of the CT/MRI slices between the studies), or even to error. In the present study, we consistently drew the renal sinus fat areas in the same way across all subjects; thus, apart from the absolute values, we think that the main results of the present study are not affected.

In conclusion, the accumulation of fat in the renal sinus is larger in patients with morbid obesity, but it is decreased following bariatric surgery. Importantly, patients with larger decreases in RSF following bariatric surgery achieved remission from hypertension, or a decrease in the number of antihypertensive drugs needed. Taken together, these findings suggest that renal sinus fat accumulation may contribute to the pathophysiology linking obesity to hypertension. 

## Figures and Tables

**Table 1:** Anthropometric and biochemical characteristics of the study participants ^§^.

	Lean	Obese		*p* Value
		*Pre*	*Post*	
N	46	74	42	-
M/W	10/36	6/68	3/39	0.03
Hypertension (N, %)	0, 0	36, 49	18, 43 *^#^	<0.0001
NGT/IFG&IGT/T2D	37/9/0	20/30/24	23/9/10 *^#^	<0.0001
Age (years)	46 ± 9	45 *±* 10	45 *±* 9	0.8
BMI (kg·m^−2^)	23.4 [21.6–24.8]	41.5 [39.1–43.9]	32.2 [29.78–34.1] *^#^	<0.0001
Systolic BP (mmHg)	123 ± 13	134 ± 17	125 ± 13 *	0.002
Diastolic BP (mmHg)	79 ± 8	86 ± 10	80 ± 9 *	<0.0001
HbA_1c_ (%)	5.5 [5.3–5.6]	5.7 [5.4–6.1]	5.6 [5.3–5.8] *	0.001
HbA_1c_ (mmol/mol)	37 [34–38]	39 [36–43]	38 [34–40] *	0.001
Plasma glucose (mmol/L)	5.4 [5.0–5.6]	5.8 [5.3–6.5]	5.3 [4.9–5.8] *	<0.0001
Plasma insulin (pmol/L)	30 [24–48]	84 [50–131]	42 [24–51] *#	<0.0001
Plasma C-peptide (nmol/l)	0.53 [0.42–0.68]	1.10 [0.87–1.40]	0.70 [0.59–0.85] *#	<0.0001
OGIS (ml·min^−1^m^−2^)	424 [387–443]	330 [278–368]	424 [369–465]	<0.0001
Total cholesterol (mmol/L)	4.5 [4.1–5.0]	4.1 [3.7–4.8]	4.1 [3.6–4.8]	0.06
LDL cholesterol (mmol/L)	2.6 [2.1–3.0]	2.6 [2.0–2.9]	2.3 [1.8–3.0]	0.8
HDL cholesterol (mmol/L)	1.6 [1.4–2.1]	1.3 [1.1–1.4]	1.4 [1.2–1.7]	<0.0001
Triglycerides (mmol/L)	0.86 ± 0.4	1.29 ± 0.47	1.0 ± 0.42 *	<0.0001
C-reactive protein (mg/L)	0.6 [0.2–1.0]	3.2 [1.8–5.3]	1.0 [0.5–2.0] *	<0.0001
Creatinine (μmol/L)	68 [60–76]	65 [58–71]	60 [52–65]	0.1
eGFR (ml/1.73 m^2^/min)	98 [93–107]	100 [87–112]	110 [91–115] *	0.5
Total eGFR (ml/min)	99 [93–108]	129 [111–140]	120 [104–129] #	<0.0001
Left RSF (cm^2^)	1.8 [1.3–2.5]	2.2 [1.6–2.9]	1.5 [1.2–2.1] *	0.0995
Right RSF (cm^2^)	1.7 [1.3–2.4]	2.5 [1.7–3.3]	1.9 [1.4–2.3] *	0.004
Average RSF (cm^2^)	1.8 [1.4–2.5]	2.3 [1.7–3.1]	1.6 [1.3–2.3] *	0.003
Total SAT (Kg)	3.9 [2.6–5.1]	17.2 [15.1–22.0]	11.0 [8.4–13.1] *^#^	<0.0001
Total VAT (Kg)	1.1 [0.7–1.7]	4.5 [3.1–5.8]	2.4 [1.4–3.4] *^#^	<0.0001

^§^ entries are mean ± SD, or median [interquartile range], as appropriate. *p* value for the comparison between obese pre and lean individuals; * *p* < 0.05 for obese before and after bariatric surgery; ^#^ *p* < 0.05 for the comparison obese post vs. lean individuals. NGT: normal glucose tolerance; IFG: impaired fasting glucose; IGT: impaired glucose tolerance; T2D: type 2 diabetes; BMI: body mass index; BP: blood pressure; OGIS: oral glucose insulin sensitivity; RSF: renal sinus fat area; eGFR: estimated glomerular filtration rate; SAT: subcutaneous adipose tissue; VAT: visceral adipose tissue.

## Data Availability

Data were available from the corresponding author, after reasonable request.
